# Characterization of *Diaporthe* species associated with peach constriction canker, with two novel species from China

**DOI:** 10.3897/mycokeys.80.63816

**Published:** 2021-05-18

**Authors:** Xianhong Wang, Yashuang Guo, Yamin Du, Ziling Yang, Xinzhong Huang, Ni Hong, Wenxing Xu, Guoping Wang

**Affiliations:** 1 Key Lab of Plant Pathology of Hubei Province, College of Plant Science and Technology, Huazhong Agricultural University, Wuhan, Hubei, 430070, China Huazhong Agricultural University Wuhan China; 2 Key Laboratory of Horticultural Crop (Fruit Trees) Biology and Germplasm Creation of the Ministry of Agriculture, Wuhan, Hubei, 430070, China Research Institute of Pomology, Fujian Academy of Agricultural Sciences Fuzhou China; 3 Research Institute of Pomology, Fujian Academy of Agricultural Sciences, Fuzhou, Fujian, 350013, China Key Laboratory of Horticultural Crop Biology and Germplasm Creation of the Ministry of Agriculture Wuhan China

**Keywords:** Constriction canker, *
Diaporthe
*, multi-gene phylogeny, *Prunus persica*, taxonomy, two new taxa

## Abstract

Species of *Diaporthe* infect a wide range of plants and live *in vivo* as endophytes, saprobes or pathogens. However, those in peach plants are poorly characterized. In this study, 52 *Diaporthe* strains were isolated from peach branches with buds, showing constriction canker symptoms. Phylogenetic analyses were conducted using five gene regions: internal transcribed spacer of the ribosomal DNA (ITS), translation elongation factor 1-α (*TEF*), ß-tubulin (*TUB*), histone (*HIS*), and calmodulin (*CAL*). These results coupled with morphology revealed seven species of *Diaporthe*, including five known species (*D. caryae*, *D. cercidis*, *D. eres*, *D. hongkongensis*, and *D. unshiuensis*). In addition, two novel species *D. jinxiu* and *D. zaofenghuang* are introduced. Except for the previously reported *D. eres*, this study represents the first characterization of *Diaporthe* species associated with peach constriction canker in China, and contributes useful data for practicable disease management.

## Introduction

The genus *Diaporthe* (asexual morph *Phomopsis*) was established by Nitschke in 1870 and predates its sexual morph established in 1905, thus *Diaporthe* is used for this genus (Rossman et al. 2015). The sexual morph of *Diaporthe* is characterized by black spherical ascomata with single or multiple tapering perithecial necks. Their asci are unitunicate, 8-spored, sessile, and elongate to clavate. The ascospores are hyaline, two-celled, often biguttulate, and elliptical to fusiform (Udayanga et al. 2015; Guo et al. 2020). The asexual morph is characterized by black or dark brown conidiomata, with cylindrical phialides producing three types of conidia (Udayanga et al. 2011; Gomes et al. 2013; Guo et al. 2020). In early studies, species of *Diaporthe* were identified based on host association, morphology and cultural characteristics (Uecker 1988). Recently, studies have shown that many species of *Diaporthe* are not host-specific i.e., one species may infect more than one host species, e.g., *D. eres* can infect blackberry (Vrandecic et al. 2011), pear (Bai et al. 2015), and jujube (Zhang et al. 2018). Moreover, a single species is prone to morphological changes depending on the incubation conditions (Gomes et al. 2013). Therefore, molecular data have been adopted to resolve the circumscription of species of *Diaporthe*, initially relying on the internal transcribed spacer (ITS) of the ribosomal DNA region (Santos et al. 2009; Thompson et al. 2011), and recently on multiple loci including ITS, translation elongation factor 1-α (*TEF*), ß-tubulin (*TUB*), histone (*HIS*), and calmodulin (*CAL*) gene regions (Santos et al. 2017). At present, the five-locus dataset (ITS-*TEF*-*CAL*-*HIS*-*TUB*) has been optimally adopted for the species delimitation by recent authors (Gao et al. 2017; Yang et al. 2018, 2020; Crous et al. 2020; Guo et al. 2020; Hyde et al. 2020; Sun et al. 2021).

Peach (*Prunus persica* L.) originated from China, where it has been cultivated for more than 3,000 years (Faust and Timon 2010). In the past ten years, the national annual production of peach and nectarine was 10–15 million tons, accounting for 50% of global production (http://www.fao.org/faostat/en/#data/QC). In recent years, peach constriction canker has been frequently observed in peach orchards in Fujian province, one of the important peach-cultivation areas in China. This disease can cause flower bud necrosis, no flowering, and even kill the shoots, resulting in a severe economic loss for growers. Peach constriction canker was firstly observed in 1934 in New Jersey, USA (Daines et al. 1958), and usually infects peach buds leading to the formation of reddish-brown elongate cankers around twig nodes, which can girdle and kill buds the following summer (Lalancette and Robison 2001). In a previous study, *P. amygdali* was identified as the cause of peach constriction canker based on morphology in Spain (Tuset et al. 1989). However, morphology alone is not adequate for determining a species in *Diaporthe*. Moreover, *Diaporthe* spp. associated with peach plants have not been well documented. To understand the etiology of peach constriction canker in China, diseased samples were collected and isolates obtained from them. The characterization of these isolates revealed five known and two novel *Diaporthe* species associated with the disease.

## Materials and methods

### Sampling and isolation

The infected peach branches with buds showing constriction canker symptoms were collected in Fujian province of China in 2017–2018. The collected samples were subjected to fungal isolation following the protocol described by Bai et al. (2015). Diseased tissues (4–5 mm^2^) were excised from infected bud scales after they were surface-sterilized with 75% ethanol for 45 s and 1% NaOCl for 45 s, rinsed twice with sterilized water, and air-dried. The excised tissues were placed on potato dextrose agar (PDA, 20% diced potatoes, 2% dextrose and 1.5% agar) plates and incubated at 25 °C in the dark for 3–5 d. After colonies grow, their mycelium was transferred to a new PDA plate and each colony was designated as a specific isolate. Each isolate was further purified by culturing from a single conidium (Choi et al. 1999). The obtained isolates were stored in 25% glycerol at -80 °C for further usage. Specimens of new species were deposited in the Mycological Herbarium, Institute of Microbiology, Chinese Academy of Sciences, Beijing, China (**HMAS**). Ex-type living cultures were deposited in the China General Microbiological Culture Collection Centre (**CGMCC**), Beijing, China. Sequences of the novel species were submitted to MycoBank (http://www.mycobank.org).

### Morphological analyses

Fungal morphology was determined by culturing a 5-d-old mycelial disc (5 mm in diameter) on a petri dish containing PDA and oatmeal agar (OA) (Udayanga et al. 2014), respectively. Cultures were incubated at 25 °C with a 14/10 h fluorescent light/dark cycle. Growth rate (mm/d) was determined by measuring the colony diameters of each isolate on PDA daily for 3 days. The colony morphologies were recorded after 14 d. Moreover, the shape, color, and size of conidiomata, conidia and conidiophore were observed using an optical microscope (Olympus BX63 or Olympus SZX16, Japan), and 50 conidia of each isolate were measured to determine their size.

### DNA extraction and determination of the taxonomic region sequences

Genomic DNA was extracted from pure culture using modified cetyltrimethyl-ammonium bromide (CTAB) protocol (Udayanga et al. 2012), and subjected to PCR amplification of the partial regions of the five loci comprised ITS, *TUB*, *TEF*, *CAL*, and *HIS* using corresponding primer pairs: ITS5/ITS4 (White et al. 1990), Bt2a/Bt2b (Glass and Donaldson 1995), EF1-728F/EF1-986R (Carbone and Kohn 1999), CAL-228F/CAL-737R (Carbone and Kohn 1999), and CYLH3F/H3-1b (Glass and Donaldson 1995, Crous et al. 2006), respectively. PCR programs were initiated with 95 °C for 5 min, followed by 34 cycles of denaturation at 95 °C for 30 s, annealing at a suitable temperature for 30 sec (56 °C for ITS, 52 °C for *TEF*, 54 °C for *CAL*, 57 °C for *HIS* and 60°C for *TUB*), and extension at 72 °C for 30 sec, and terminated with a final elongation step at 72 °C for 10 min.

The PCR amplicons were purified and sequenced at the Sangon Biotech (Shanghai, China) Company. Consensus sequences were obtained using DNAMAN (v. 9.0, Lynnon Biosoft), and deposited in GenBank (Suppl. material 1: Table S1).

### Phylogenetic analyses

Sequences generated in this study were blasted against the NCBI GenBank nucleotide database to determine the closest relatives. Alignment of different gene regions of isolates obtained in this study, their relatives and the ones of the type species (Suppl. material 2: Table S2) was initially performed using the MAFFT v. 7 online servers (http://mafft.cbrc.jp/alignment/server/index.html) (Katoh and Standley 2013) with default settings, and the alignment was manually adjusted in MEGA v. 7 (Kumar et al. 2016).

Phylogenetic analyses were conducted based on the concatenated five loci. Bayesian inference (BI) was used to construct phylogenies using MrBayes v. 3.1.2 (Ronquist and Huelsenbeck 2003). The best-fit model of nucleotide substitution for each partition was determined using MrModeltest v. 2.3 (Nylander 2004) and incorporated into the analyses. Two analyses of four Markov Chain Monte Carlo (MCMC) chains were conducted from random trees with 1.8 × 10^8^ generations. The analyses were sampled every 1,000 generations, which were stopped once the average standard deviation of split frequencies was below 0.01. The first 25% of the trees were discarded as the burn-in phase of each analysis, and the remaining trees were summarized to calculate the posterior probabilities (PP) of each monophyletic clade.

Additionally, maximum parsimony analyses (MP) were performed on the multi-locus alignment using PAUP (Phylogenetic Analysis Using Parsimony) v. 4.0b10 (Swofford 2002). Phylogenetic trees were generated using the heuristic search option with Tree Bisection Reconnection (TBR) branch swapping and 1,000 random sequence additions. Max trees were set up to 5,000, zero-length branches were collapsed, and all multiple parsimonious trees were saved. Clade stability was assessed using a bootstrap analysis with 1,000 replicates. Tree length (TL), consistency index (CI), retention index (RI), rescaled consistency index (RC), and homoplasy index (HI) were calculated. Furthermore, maximum likelihood (ML) analysis was performed using IQtree v. 1.6.8. The analysis was performed with a GTR site substitution model. Branch support was evaluated with a bootstrapping (BS) method of 1,000 replicates (Hillis and Bull 1993). Phylogenetic trees were visualized in FigTree v. 1.4.2 (Rambaut 2014). The alignments and phylogenetic trees were deposited in TreeBASE (submission ID: 28014).

## Results

### Collection of *Diaporthe* strains

**Figure 1.**
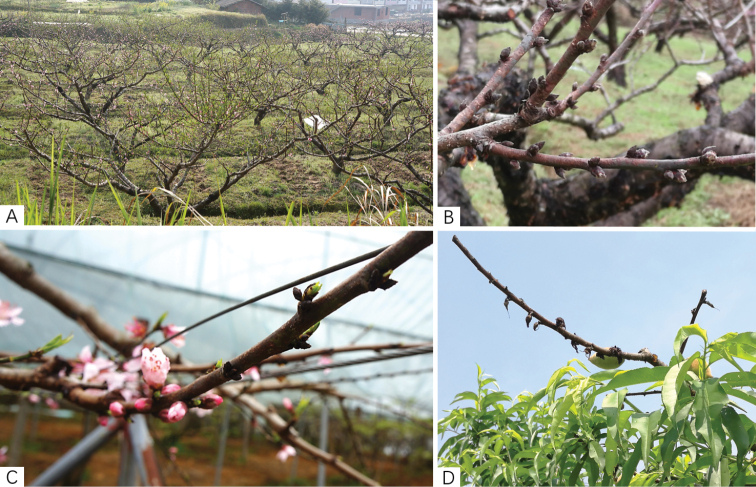
Representative symptoms of peach constriction canker in the field **A** blossom bud necrosis, few or no flowers on peach trees in spring **B** black-brown lesions around buds **C** petrified blossom buds **D** rigid leaf buds.

In the surveyed orchards, this disease caused flower-bud necrosis, little or no flowering (Fig. 1A), black-brown lesions around the buds (Fig. 1B), petrified blossom buds (Fig. 1C), and rigid leaf buds (Fig. 1D). A total of 52 *Diaporthe* strains were obtained from 27 infected bud samples of *Prunus persica* cultivars (cvs.) Baifeng, Jinxiu, Jinyuan, and Zaofenghuang by fungal isolation and single spore culturing. All strains were used in morphological observation and phylogenetic analysis.

### Phylogenetic analyses

**Figure 2.**
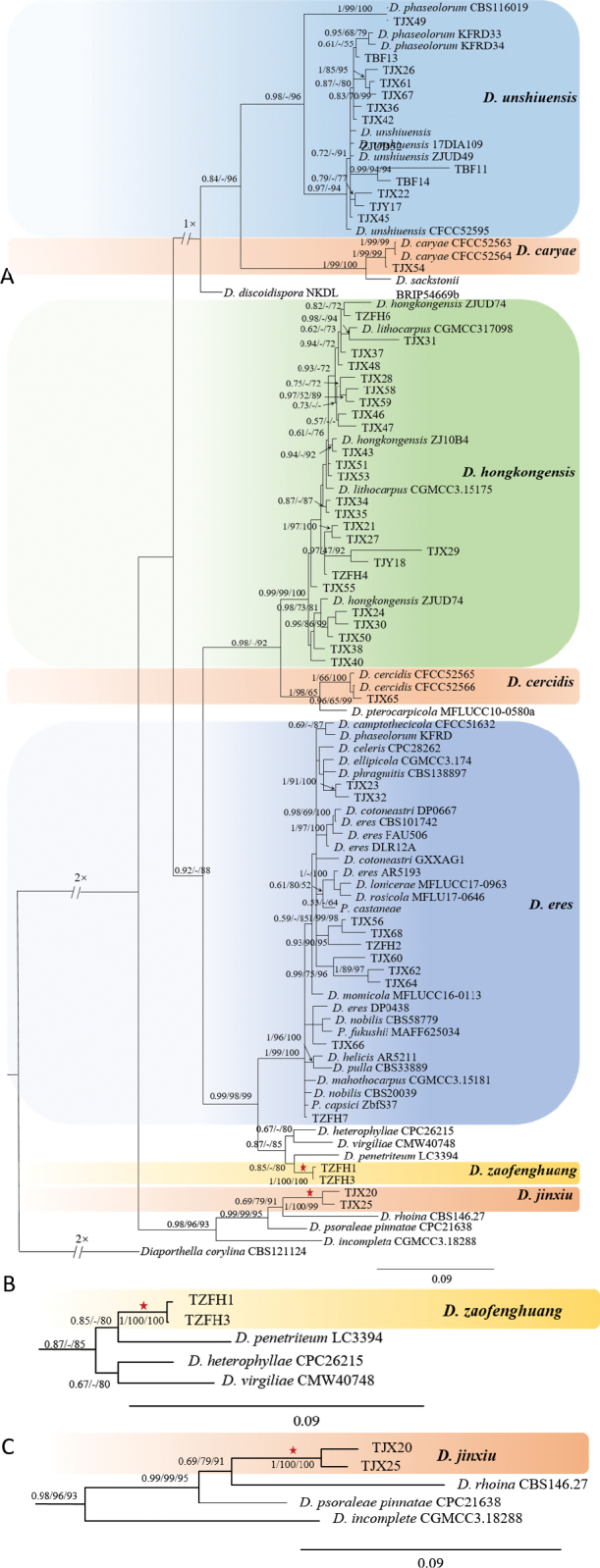
**A** phylogenetic tree generated by Bayesian analysis based on combined ITS, *TEF*, *CAL*, *HIS*, and *TUB* sequence. *Diaporthella corylina* (CBS121124) was selected as the outgroup. Bayesian posterior probability (PP ≥ 0.90), MP bootstrap support values (MP ≥ 50%) and RAxML bootstrap support values (ML ≥ 50%) are shown at the nodes (PP/ML/MP). The branches of the new *Diaporthe* species are marked with red stars **B, C** are partial phylogenetic taxa highlighting *D. zaofenghuang* and *D. jinxiu* together with their closely related species, respectively.

The strains mentioned above together with 49 reference isolates of previously described species (Suppl. material 2: Table S2) were subjected to multi-locus phylogenetic analyses with concatenated ITS, *TEF*, *CAL*, *HIS*, and *TUB* sequences. *Diaporthella corylina* (CBS 121124) was selected as the outgroup. A total of 2,514 characters of nucleotides and gaps (ITS: 1–578, *TEF*: 579–960, *CAL*: 961–1,469, *HIS*: 1,470–1,989, *TUB*: 1,990–2,514) were included in the phylogenetic analysis. The maximum-likelihood (ML) tree was generated with the GTR model. The best nucleotide substitution models were recommended by MrModeltest and used in the Bayesian analysis: SYM+I+G for ITS, GTR+G for *TEF*, HKY+I+G for *TUB*, and GTR+I+G for both *CAL* and *HIS*. The heuristic search using MP generated 1,000 parsimonious trees (TL = 3,182, CI = 0.52, RI = 0.87, RC = 0.45), and branches of zero length were collapsed and all multiple parsimonious trees were saved. MP and ML bootstrap support values above 50% are shown as second and third position above the nodes, respectively. In the phylogenetic tree, 48 of the 52 isolates obtained in this study were assigned to five known species, *D. caryae* (one strain), *D. cercidis* (one), *D. eres* (nine), *D. hongkongensis* (26), and *D. unshiuensis* (11). Four strains formed two distinct clades, and were identified as two novel species, described herein as *D. zaofenghuang* (two strains, closely related to *D. penetriteum*), and *D. jinxiu* (two strains, close to *D. rhoina*), respectively (Fig. 2).

## Taxonomy

### Diaporthe jinxiu

Taxon classificationFungiDiaporthalesDiaporthaceae

X.H. Wang & G.P. Wang sp. nov.

62B030DF-B3AB-5F15-9BBA-6FC2E23CD972

838502

Fig. 3

#### Etymology.

Named for the host variety (*Prunus persica* cv. Jinxiu), from which the species was isolated.

#### Description.

Sexual morph: not observed. Asexual morph on alfalfa stems after 15 days. *Pycnidial conidiomata* small, covered by pale yellow discharged conidial masses at maturity, 385–810 μm diam. *Conidiophore* hyaline, cylindrical, smooth, phialidic, unbranched, straight or slightly curved, 16–21 × 2–2.5 μm. *Conidiogenous cells* phialidic, cylindrical. Alpha *conidia* hyaline, aseptate, ellipsoidal, biguttulate, rounded at each end, 5.8–7.1 × 2.7–4.0 µm (mean = 6.4 ± 0.4 × 3.5 ± 0.3 μm, n = 50). *Beta* and *gamma conidia* not observed.

**Culture characteristics.** Cultures incubated on PDA at 25 °C in cycle of light/darkness, growth rate 11.5 mm per day. On PDA medium, colonies were sparse and villous, flourishing at edge of colony. On OA medium, colonies dense with neat edges, with yellow pigment in the center.

#### Materials examined.

China, Fujian Province, Sanming City, on buds of *Prunus persica* cv. Zaofenghuang, 23 March 2017, Y. S. Guo (holotype HMAS 249837, culture ex-holotype culture CGMCC3.20269 = TZFH20); ibid., ex-isotype culture TZFH25.

#### Notes.

In the phylogenetic, multi-locus tree, *D. jinxiu* forms a distinct clade with maximum support (1/100/99) and is most closely related to *D. rhoina*, but with smaller pycnidial conidiomata than the later (*D. jinxiu* = 386–807 μm vs *D. rhoina* = 500–2500 μm) (Feltgen 1901). Moreover, the sequence differences were significant, and all five regions were able to distinguish them (28/578 in ITS, 38/382 in *TEF*, 21/509 in *CAL*, 28/520 in *HIS*, and 11/525 in *TUB*).

**Figure 3.**
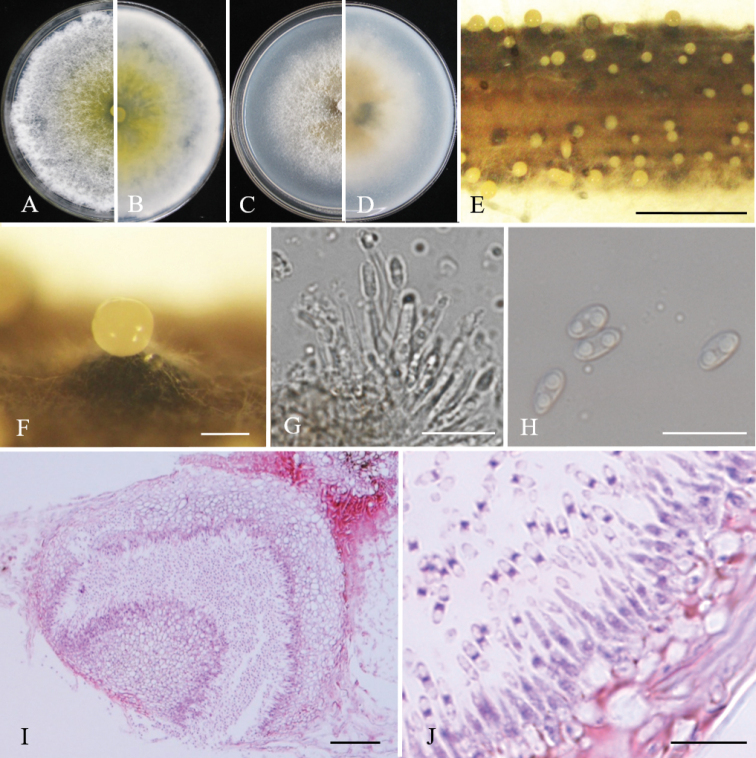
*Diaporthe jinxiu*. Front and back views of colonies on PDA (**A, B**) and OA (**C, D**), respectively **E** conidiomata on alfalfa stems **F** conidioma **G** conidiophores **H***alpha* conidia **I–J** section view of conidioma. Scale bars: 2 mm (**E**); 200 μm (**F**); 20 μm (**I**); 10 μm (**G, H, J**).

### Diaporthe zaofenghuang

Taxon classificationFungiDiaporthalesDiaporthaceae

X.H. Wang & G.P. Wang sp. nov.

0AD93BED-5368-5375-8F26-6C5AC411C956

838501

Fig. 4

#### Etymology.

Named after the host species (*Prunus persica* cv. Zaofenghuang) from which the species was isolated.

#### Description.

Sexual morph not observed. Asexual morph on alfalfa stems. *Pycnidial conidiomata* conical, yellowish translucent conidial drops exuded from ostioles, 650–1430 μm diam. *Conidiophores* fasciculate, hyaline, long cylindrical, straight or slightly curved, apex pointed, 13.7–20.9 × 1.8–2.7 μm. *Conidiogenous cells* phialidic, cylindrical. *Alpha conidia* hyaline, aseptate, ellipsoidal, biguttulate, rounded at one end, slightly apex at another end, 5.3–7.5 × 2.9–3.7 µm (mean = 6.0 ± 0.6 × 3.1 ± 0.3 μm, n = 50). *Beta* and *gamma conidia* not observed.

#### Culture characteristics.

Cultures incubated on PDA at 25 °C in cycle of light/darkness, growth rate 8.5 mm per day. Colonies initially white on surface, producing black pigment from center of medium and expanding outwardly after 5–7 d. On PDA, edge of colony petal-like, irregular; on OA, edge relatively flat.

#### Materials examined.

China, Fujian Province, Sanming City, on buds of *Prunus persica* cv. Zaofenghuang, 23 March 2017, Y. S. Guo (holotype HMAS 249835, culture ex-holotype CGMCC3.20271 = TZFH1); ibid., culture TZFH3.

#### Notes.

Two isolates representing *D. zaofenghuang* form a well-supported clade (1/100/100) and appear to be most closely related to *D. penetriteum*. *Diaporthe zaofenghuang* can be distinguished from *D. penetriteum* based on ITS, *HIS*, and *TUB* loci (10/578 in ITS, 45/520 in *HIS*, and 7/525 in *TUB*). Morphologically, *D. zaofenghuang* differs from *D. penetriteum* in having larger conidiomata (*D. zaofenghuang* = 653–1433 μm vs *D. penetriteum* = 180–490 μm) and *alpha* conidia (*D. zaofenghuang* = 6.0 ± 0.6×3.1 ± 0.3 μm vs *D. penetriteum* = 5.0 ± 0.3 × 2.2 ± 0.2 μm). Additionally, *D. penetriteum* produces two types of conidia, but *D. zaofenghuang* produces only *alpha* conidia (Gao et al. 2016).

**Figure 4.**
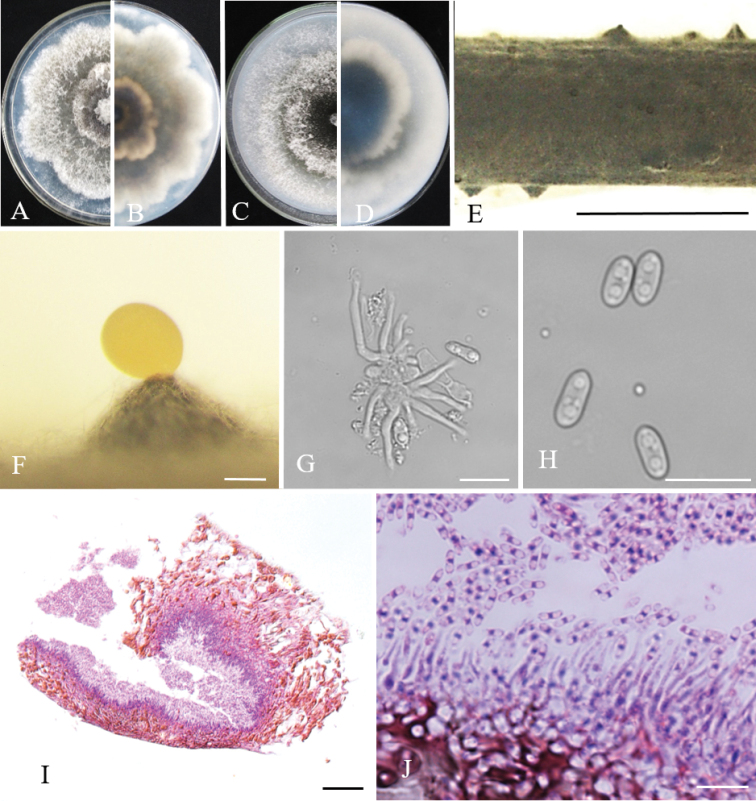
*Diaporthe zaofenghuang*. Front and back views of colonies on PDA (**A, B**) and OA (**C, D**), respectively **E** conidiomata on alfalfa stems **F** conidioma **G** conidiophores **H***alpha* conidia **I–J** section view of conidioma. Scale bars: 2 mm (**E**); 200 μm (**F**); 20 μm (**G, I**); 10 μm (**H, J**).

## Discussion

In this study, phylogenetic analyses based on the five combined loci (ITS, *TEF*, *CAL*, *HIS*, and *TUB*) coupled with morphology revealed seven *Diaporthe* species (viz. *D. caryae*, *D. cercidis*, *D. eres*, *D. hongkongensis*, *D. jinxiu*, *D. unshiuensis*, and *D. zaofenghuang*) associated with peach constriction canker. Of these species, two novel species *D. jinxiu* and *D. zaofenghuang* in distinct clades were described. *Diapothe jinxiu* has smaller conidiomata as compared to its closest relative *D. rhoina*. However, it is not possible to have more morphological comparisons at this stage because limited biological information is available for this species (Feltgen 1901). Moreover, *D. jinxiu* clearly differs from other phylogenetically related species, e.g., *D. psoraleae-pinnatae*, by having shorter and wider *alpha* conidia (*D. jinxiu* = 5.8–7.1 × 2.7–4.0 μm vs *D. psoraleae-pinnatae* = 7.0–12.0 × 2.5–3.0 μm) (Crous et al. 2013), and yellow pigments (Fig. 3A–D). *Diaporthe zaofenghuang* showed different morphologies compared with *D. penetriteum*, e.g., larger *alpha* conidia, larger conidiomata, and no *beta* conidia (Gao et al. 2016). Therefore, both novel species described here are clearly separated from known ones in the phylogeny and morphology.

Based on molecular data, several *Diaporthe* species associated with peach diseases in other countries have been identified and characterized, including *D. amygdali*, which is responsible for apical dead shoot, twig and shoot blight in peach and nectarine in Uruguay (Sessa et al. 2017), and *D. eres* for stem canker on peach in Italy and Greece (Thomidis et al. 2009; Prencipe et al. 2017). *Diaporthe amygdali* was not found in the present study but Dai et al. (2012) recorded it as *Phomopsis amygdali* related to twig canker of peach. Previously, six *Diaporthe* spp. infecting peach trees have been characterized in China. The reported *D. hongkongensis* caused fruit rot (Zhang et al. 2021), *D. eres*, *D. momicola*, *D. pescicola*, and *D. taoicola* were related to tree dieback (Dissanayake 2017), and *D. amygdali* was related to twig canker (Dai et al. 2012). To our knowledge, besides *D. eres*, this is the first report of *D. caryae*, *D. cercidis*, *D. hongkongensis*, *D. jinxiu*, *D. unshiuensis*, and *D. zaofenghuang* as the cause of peach constriction canker. This study contributes useful information for practicable disease management.

Previous studies have revealed that species of *Diaporthe* are highly divergent and closely linked to sampling areas, such as 19 species of *Diaporthe* infecting pears cultivated in 15 provinces of China (Guo et al. 2020). Further studies are required to include an extensive collection of *Diaporthe* isolates from other peach-cultivated regions in China. For effective disease management, more knowledge is required about *Diaporthe* species related to peach constriction canker in China.

## Supplementary Material

XML Treatment for Diaporthe jinxiu

XML Treatment for Diaporthe zaofenghuang

## Supplementary materials

Supplementary material 1 GenBank accession numbers of isolates included in this study Data type GenBank accession numbers Explanation note GenBank accession numbers of sequences obtained from this study. https://binary.pensoft.net/file/544344This dataset is made available under the Open Database License (http://opendatacommons.org/licenses/odbl/1.0/). The Open Database License (ODbL) is a license agreement intended to allow users to freely share, modify, and use this Dataset while maintaining this same freedom for others, provided that the original source and author(s) are credited.Author: Xianhong Wang

Supplementary material 2 List of *Diaporthe* species used to phylogenetic analysis in this study, with details about host, country, and GenBank accession numbers Data type GenBank accession numbers Explanation note GenBank accession numbers of sequences downloaded from GenBank. https://binary.pensoft.net/file/544345This dataset is made available under the Open Database License (http://opendatacommons.org/licenses/odbl/1.0/). The Open Database License (ODbL) is a license agreement intended to allow users to freely share, modify, and use this Dataset while maintaining this same freedom for others, provided that the original source and author(s) are credited.Author: Xianhong Wang
