# Supplementary material for: Characterization of Diaporthe species associated with peach constriction canker, with two novel species from China
Source: MycoKeys. 2021 May 18;80:77–90. doi: 10.3897/mycokeys.80.63816 (PMC8149378; doi:10.3897/mycokeys.80.63816)
Supplement: Supplementary material 1 — GenBank accession numbers of isolates included in this study [file mycokeys-80-077-s001.docx]

Supplementary Table 1 GenBank accession numbers of isolates included in this study

| **Species** | **Culture no.** | **GenBank accession number** | | | | | |
| --- | --- | --- | --- | --- | --- | --- | --- |
|  |  | **ITS** | | ***CAL*** | ***HIS*** | ***TEF*** | ***TUB*** |
| *D. caryae* | TJX54 | MW485743 | MW350723 | | MW491971 | – | MW368970 |
| *D. cercidis* | TJX65 | MW341306 | MW491970 | | – | MW362968 | MW491981 |
| *D. eres* | TZFH2 | MW341259 | MW491961 | | MW350724 | MW362923 | MW362969 |
|  | TZFH7 | MW341260 | MW491962 | | MW491972 | MW362924 | MW491982 |
|  | TJX23 | MW341261 | MW362884 | | MW350725 | MW362930 | MW362970 |
|  | TJX32 | MW341262 | MW362885 | | MW350726 | MW362925 | MW362971 |
|  | TJX56 | MW341263 | MW491963 | | MW350727 | MW362926 | MW362972 |
|  | TJX60 | MW341265 | MW362886 | | MW362889 | MW362931 | MW362974 |
|  | TJX62 | MW341266 | MW362887 | | MW362890 | MW362928 | MW362975 |
|  | TJX64 | MW341267 | MW362888 | | MW362891 | MW362929 | MW362976 |
|  | TJX66 | – | MW491965 | | MW491973 | MW362922 | – |
|  | TJX68 | MW341264 | MW491964 | | MW350728 | MW362927 | MW362973 |
| *D. hongkongensis* | TZFH4 | MW341268 | MW350690 | | MW362892 | MW362932 | MW368938 |
|  | TZFH6 | MW341269 | MW350691 | | MW491974 | MW362933 | MW368939 |
|  | TJY18 | MW485729 | MW350704 | | MW362903 | MW362956 | MW368952 |
|  | TJX21 | MW341283 | MW350702 | | MW362902 | MW362954 | MW368950 |
|  | TJX24 | MW485730 | MW350706 | | MW362908 | MW362955 | MW368954 |
|  | TJX27 | MW341284 | MW350703 | | MW362910 | MW362947 | MW368951 |
|  | TJX28 | MW341273 | MW350695 | | MW491975 | MW362937 | MW368943 |
|  | TJX29 | MW341285 | – | | MW491978 | MW362948 | – |
|  | TJX30 | MW341289 | MW350707 | | MW362909 | MW362950 | MW368955 |
|  | TJX31 | MW341270 | MW350692 | | MW362893 | MW362934 | MW368940 |
|  | TJX34 | MW341281 | MW491966 | | MW491977 | MW362945 | – |
|  | TJX35 | MW341282 | MW350701 | | MW362901 | MW362946 | MW368949 |
|  | TJX37 | MW341271 | MW350693 | | MW491979 | MW362935 | MW368941 |
|  | TJX38 | MW341291 | MW350708 | | MW362905 | MW362952 | MW368957 |
|  | TJX40 | MW341292 | MW350709 | | MW362906 | MW362953 | MW368958 |
|  | TJX43 | MW341278 | MW350699 | | MW362899 | MW362942 | MW368947 |
|  | TJX46 | MW341276 | MW350710 | | MW362897 | MW362940 | MW368945 |
|  | TJX47 | MW341277 | MW350698 | | MW362898 | MW362941 | MW368946 |
|  | TJX48 | MW341272 | MW350694 | | MW362894 | MW362936 | MW368942 |
|  | TJX50 | MW341290 | MW350711 | | MW362904 | MW362951 | MW368956 |
|  | TJX51 | MW341279 | MW491968 | | MW491976 | MW362943 | – |
|  | TJX53 | MW341280 | MW350700 | | MW362900 | MW362944 | MW368948 |
|  | TJX55 | MW485731 | MW350705 | | MW362907 | MW362949 | MW368953 |
|  | TJX58 | MW341274 | MW350696 | | MW362895 | MW362938 | MW368959 |
|  | TJX59 | MW341275 | MW350697 | | MW362896 | MW362939 | MW368944 |
| *D. jinxiu* | TJX20 | MW477881 | MW480869 | | MW480865 | MW480873 | MW480877 |
|  | TJX25 | MW477882 | MW480870 | | MW480866 | MW480874 | MW480878 |
| *D. unshiuensis* | TBF11 | MW341293 | MW350712 | | MW362911 | MW362964 | MW368969 |
|  | TBF13 | MW341294 | MW350713 | | MW362912 | MW362963 | MW368967 |
|  | TBF14 | MW341295 | MW350714 | | MW362913 | MW362965 | MW368960 |
|  | TJY17 | MW341296 | MW350715 | | MW362918 | MW362966 | MW368961 |
|  | TJX22 | MW341297 | MW350716 | | MW362914 | MW362967 | MW368962 |
|  | TJX26 | MW341298 | MW350717 | | MW362920 | MW362957 | MW368966 |
|  | TJX36 | MW341299 | MW350718 | | MW362919 | MW362958 | MW368968 |
|  | TJX42 | MW341300 | MW350719 | | MW362915 | MW362959 | MW368965 |
|  | TJX45 | MW341301 | MW350720 | | MW362916 | MW362960 | MW368963 |
|  | TJX49 | MW485732 | MW350722 | | MW362921 | – | MW368971 |
|  | TJX61 | MW341302 | MW350721 | | MW362917 | MW362961 | MW368964 |
|  | TJX67 | MW341303 | MW491969 | | MW491980 | MW362962 | – |
| *D. zaofenghuang* | TZFH1 | MW477883 | MW480867 | | MW480863 | MW480871 | MW480875 |
|  | TZFH3 | MW477884 | MW480868 | | MW480864 | MW480872 | MW480876 |
