# Supplementary material for: Characterization of Diaporthe species associated with peach constriction canker, with two novel species from China
Source: MycoKeys. 2021 May 18;80:77–90. doi: 10.3897/mycokeys.80.63816 (PMC8149378; doi:10.3897/mycokeys.80.63816)
Supplement: Supplementary material 2 — List of Diaporthe species used to phylogenetic analysis in this study, with details about host, country, and GenBank accession numbers [file mycokeys-80-077-s002.docx]

Supplementary Table 2 List of *Diaporthe* species used to phylogenetic analysis in this study, with details about host, country, and GenBank accession numbers

| **Species** | **Culture no.** | **Host** | **Country** | **GenBank accession number** | | | | | |
| --- | --- | --- | --- | --- | --- | --- | --- | --- | --- |
|  |  |  |  | **ITS** | | ***CAL*** | ***HIS*** | ***TEF*** | ***TUB*** |
| *D. camptothecicola* | CFCC 51632 | *Camptotheca acuminata* | China | KY203726 | KY228877 | | KY228881 | KY228887 | KY228893 |
| *D. caryae* | CFCC 52563* | *Carya illinoensis* | China | MH121498 | MH121422 | | MH121458 | MH121540 | MH121580 |
|  | CFCC 52564 | *Carya illinoensis* | China | MH121499 | MH121423 | | MH121459 | MH121541 | MH121581 |
| *D. celeris* | CPC 28262 | *Vitis vinifera* | Czech Republic | MG281017 | MG281712 | | MG281363 | MG281538 | MG281190 |
| *D. cercidis* | CFCC 52565* | *Cercis chinensis* | China | MH121500 | MH121424 | | MH121460 | MH121542 | MH121582 |
|  | CFCC 52566 | *Cercis chinensis* | China | MH121501 | MH121425 | | MH121461 | MH121543 | MH121583 |
| *D. cotoneastri* | DP0667 | *Juglans cinerea* | USA | KC843328 | KC843155 | | – | KC84312 | KC843229 |
| *D. ellipicola* | CGMCC 3.17084* | *Lithocarpus glabra* | China | KF576270 | – | | – | KF576245 | KF576294 |
| *D. eres* | AR5193* | *Ulmus* sp. | Germany | KJ210529 | KJ434999 | | KJ420850 | KJ210550 | KJ420799 |
|  | CBS 101742 | *Fraxinus* sp. | Netherlands | KC343073 | KC343315 | | KC343557 | KC343799 | KC344041 |
|  | DLR12A | *Vitis vinifera* | France | KJ210518 | KJ434996 | | KJ420833 | KJ210542 | KJ420783 |
|  | DP0438 | *Ulmus minor* | Netherlands | KJ210532 | KJ435016 | | KJ420886 | KJ210553 | KJ420816 |
|  | FAU506 | *Cornus florida* | USA | KJ210526 | KJ435012 | | KJ420842 | JQ807403 | KJ420792 |
| *D. helicis* | AR5211= CBS 138596* | *Hedera helix* | France | KJ210538 | KJ435043 | | KJ420875 | KJ210559 | KJ420828 |
| *D. heterophyllae* | CPC 26215 | *Acacia heterophylla* | France | MG600222 | MG600218 | | MG600220 | MG600224 | MG600226 |
| *D. hongkongensis* | ZJ10B4* | *Dichroa febrífuga* | China | KC343119 | KC343361 | | KC343603 | KC343845 | KC344087 |
|  | ZJUD74 | *Citrus unshiu* | China | KJ490609 | – | | – | KJ490488 | KJ490430 |
| *D. incompleta* | CGMCC 3.18288* | *Camellia sinensis* | China | KX986794 | KX999289 | | KX999265 | KX999186 | KX999226 |
| *D. lithocarpus* | CGMCC 317098 | *Lithocarpus glaber* | China | KF576276 | KF576228 | | – | KF576251 | KF576300 |
|  | CGMCC 3.15175 * | *Lithocarpus glaber* | China | KC153104 | KF576236 | | – | KC153095 | KF576311 |
| *D. lonicerae* | MFLUCC 17-0963 | *Lonicera sp.* | Italy | KY964190 | – | | – | KY964146 | KY964073 |
| *D. mahothocarpus* | CGMCC 3.15181* | *Lithocarpus glabra* | China | KC153096 | KT459461 | | – | KC153087 | KF576312 |
| *D. momicola* | MFLUCC 16-0113 | *Prunus persica* | China | KU557563 | KU557611 | | – | KU557631 | KU557587 |
| *D. nobilis* | CBS 200.39 | *Laurus nobilis* | Germany | KC343151 | KC343393 | | KC343635 | KC343877 | KC344119 |
|  | CBS 587.79 | *Pinus pantepella* | Japan | KC343153 | KC343395 | | KC343637 | KC343879 | KC344121 |
| *D. penetriteum* | LC3394 | *Camellia sinensis* | China | KP26789 |  | | KP293544 | KP267967 | KP293473 |
| *D. phaseolorum* | CBS 116019 | *kiwifruit; variety JinYan* | China | – | KC343417 | | KC343659 | KC343901 | KC344143 |
| *D. phragmitis* | CBS 138897* | *Phragmites australis* | China | KP004445 | – | | KP004503 | – | KP004507 |
| *D. pterocarpicola* | MFLUCC 10-0580a* | *Pterocarpus indicus* | Thailand | JQ619887 | JX197433 | | – | JX275403 | JX275441 |
| *D. psoraleae pinnatae* | CPC 21638 | *Psoralea pinnata* | South Africa | KF777159 | – | | – | – | KF777252 |
| *D. pulla* | CBS 338.89* | *Hedera helix* | Yugoslavia | KC343152 | KC343394 | | KC343636 | KC343878 | KC344120 |
| *D. rhoina* | CBS 146.27 | *Toxicodendron pubescens* |  | KC343189 | KC343431 | | KC343673 | KC343915 | KC344157 |
| *D. rosicola* | MFLU 17-0646 | *Rosa sp.* | *United Kingdom* | MG828895 | MG829274 | | – | MG829270 | MG843877 |
| *D. sackstonii* | BRIP 54669b* | *Helianthus annuus* | Australia | KJ197287 | – | | – | KJ197249 | KJ197267 |
| *D. unshiuensis* | ZJUD52* | *Citrus* sp. | China | KJ490587 | – | | KJ490529 | KJ490466 | KJ490408 |
|  | ZJUD49 | *Citrus* sp. | China | KJ490584. | – | | KJ490526 | KJ490463 | KJ490405 |
|  | CFCC 52595 | *Carya illinoinensis* | China | MH121530 | – | | MH121488 | MH121572 | MH121607 |
| *D. virgiliae* | CMW40748 | *Virgilia oroboides* | South Africa | KP247566 | – | | – | – | KP247575 |
| *P. capsici* | Zbf-S37 | *Zanthoxylum bungeanum* |  | KX065028 | – | | – | – | – |
| *P. castaneae* |  | *Castanea mollissima* |  | JF957786 | – | | – | – | – |
| *P. fukushii* | MAFF625034 | *Pyrus pyrifolia* | Japan | JQ807469 | – | | – | JQ807418 | – |
| *Diaporthella corylina* | CBS 121124* | *Corylus* sp. | China | KC343004 | KC343246 | | KC343488 | KC343730 | KC343972 |

* = Ex-type culture.
